# Remarkable Cases of Madness

**Published:** 1802-10-01

**Authors:** John Vaughan

**Affiliations:** Wilmington


### *Remarkable Cases of Madness; communicated by Dr.* JOHN VAUGHAN, *of Wilmington.*

[From the Medical Repoſitory, edited by Drs. Mitchell and Miller, and publiſhed at New York.]

IN preſenting you with the following account of a family-mania, I am flattered with the belief, that it will be ranked amongſt the wonders of the nineteenth century. When witchcraft and conjuration ſwayed the ſceptre of public opinion, the narration of this fact might not have excited much attention: but in the preſent day it will be deemed an intereſting evidence of the frailty of the human mind.

The family in which this infectious mania occurred live a few miles north-weſt of this borough, and have long been eſteemed induſtrious, orderly people, until Auguſt laſt, when they became infatuated with the notion of being *possessed with an evil spirit.*—Their phrenzy is thus deſcribed by a reſpectable farmer, who was an obſerver of a part of the tragedy:

"On the 3d of Auguſt, 1801, S— S— came to his mother's (who had been inſane for ſome time) to ſettle ſome buſineſs with her, when ſhe caught him round the neck, and kiſſed him, telling him he ſhould become a preacher of the everlaſting goſpel, when *he* immediately became crazy, and thought he was inſpired. On the evening of the next day ſhe kiſſed him again, and alſo kiſſed two other ſons, two daughters, and two daughters-in-law, and the whole of them became frantic immediately.

"On the morning of the 5th I was ſent for in great haſte, when I found the whole family in the utmoſt confuſion, believing they were poſſeſſed with an evil ſpirit; adding, that their mother had died a week before, and Satan had entered into her body, and communicated himſelf to the reſt of them by *a kiss!* Under this impreſſion, they had dragged the old woman out of bed, and nearly beaten her to death. After a conſiderable ſtruggle I reſcued her from them, and laid her in bed. They then en-

 deavoured deavoured to ſet the houſe on fire, to conſume the tormenting demon in the image of their mother; but ſeveral of the neighbours collecting, we ſeparated them, and prevented further miſchief. In the courſe of a few days they all became peaceable and rational but John, who afterwards became your patient.

"WILLIAM SIMONSON."

Of the truth of theſe circumſtances, related by Mr. Simonſon, there can be no doubt. I have, for many years, known him to be a man of unqueſtionable integrity, and highly reſpected in his neighbourhood, and, as he lived within a few rods of the infected family, I applied to him for an account of the fact.

John S—, who is mentioned as becoming my patient, remained iraſcible after the recovery of his brothers and ſiſters, and was confined in the Paupers' Infirmary for a few days, where he was freely blooded without relief; and on the tenth day of his diſeaſe he was brought to the houſe of a relation in this town.

I was called to ſee him on the following day, when I found him chained to the floor, with his hands tied acroſs his breaſt —cloaths torn off, except his ſhirt—his feet and elbows bruiſed conſiderably—and his countenance, grimaces, and incoherent language, truly deſcriptive of his unhappy condition. As he was free from fever, and his pulſe not tenſe nor preternaturally full, I deemed this a fair caſe for the application of cold water, as recommended by Dr. G. G. Brown, in *apoplexia mentalis*, or *delirium sine febre*. (See Med. Rep. vol. iv. p. 209.) Accordingly a linen cloth was wrapped round the head, and wetted every few minutes, by a ſponge, with cold water, for five hours, without any ſenſible effect.

On the twelfth morning of the diſeaſe it was reſumed, and continued until it induced chillineſs; and as it produced no relief, his friends became impatient of a remedy apparently ſimple; and on the next day his head was ſhaved, ſixteen ounces of blood taken from the arm, and a cathartic exhibited. The ſucceeding night he ſlept tolerably well, but remained incoherent; and on the fourteenth morning a bliſter was applied to the ſcalp, and the purge renewed, which relieved him ſo far as to enable him to go home, and attend to the buſineſs of his farm. He, however, retained ſome idea of his previous deluſion, and believed he was commiſſioned to be a preacher, and entitled to ſupport from the public; but the infatuation gradually wore away, and he is now perfectly reſtored.

The reſt of the family remain well. The old woman *actually* died in a ſhort time after the melancholy ſcene of confuſion before-mentioned.

This

This cafe affords ample fource of fpeculation to the metaphyfical pathologift who wifhes to explain the morbid affections of the mind and body. For my part, I confefs myfelf much at a lofs to attempt any explanation of this extraordinary form of mania, independent of any previous bodily difeafe. Dr. Rufh denominates mania a ftate of fever, but I prefume the prefent affection is an exception to that doctrine.

It may be obferved, that the mother had been, for fome months, in a chronic ftate of infanity; and as her children, no doubt, were much interefted in her condition, their affections wrought up, at this juncture, by a final fettlement of bufinefs which impreffed them with apprehenfions of her being irrecoverable, together with the predifpofing force of prejudice and credulity, may have favoured the fanatical impreffion.

I find, on a minute inquiry of fome of the connections of the family, that the whole of them embraced the idea of John's being commiffioned to preach the gofpel, and they accordingly initiated him in the functions of a minifter, by the performance of the ceremonials of their church.

That phyfical fomething, ufually ftiled fympathy, which is confidered as the fource of compaffion, which is excited into action by the fight of our fellow creatures in diftrefs, may be fufceptible of morbid influence beyond fatisfactory explanation. It is well known that convulfive affections have been induced by the fight of perfons labouring under them, and heroes have been known to weep at the impreffive fpectacles of deftruction made by their own victorious arms, on the reflection that fuch might be the fate of their own country, or their own friends.

The phrafes, *sympathetic action*, *consent of action*, and *associated action*, are becoming familiar in medical language; but, are they not rather medical expreffions ufed to illuftrate a connection or relationfhip between the different parts of the fyftem, inftead of defignating any peculiar law of the animal economy? We find that fympathetic or affociated actions vary with the ftates of the fyftem. Peftilential miafmata will at one time excite vomiting; at another time mufcular debility, or languor; and, perhaps, a third time febrile action; and are frequently inefficient in producing difeafe of any kind, from the non-concurrence of a predifpofing caufe. Thus alfo are mental impreffions varied by incidental ftates of the fyftem. At fome times the mere found of death will excite horror, and at other times the devaftations of peftilence are viewed with as much indifference as if we were invulnerable. And, upon an extenfive view of the difpofitions and actions of mankind, it would feem as if fome perfons were conftitutionally devoid of fympathy,

ſympathy, and thoſe tender affections which ennoble the character of man; or, from an unfavourable hebetude, are inſenſible to thoſe impreſſions which frequently torture the minds of more ſuſceptible beings. Therefore, prediſpoſition of body, and ſympathy of mind, probably deſerve an equal rank in the formation of corporeal and mental diſeaſes.

Admitting the ſtomach to be the centre of aſſociation in the eſtabliſhment of febrile diſeaſes, may not the *senſorium commune* be entitled the centre of aſſociation in mental diſorders? The connection of the animal functions, and their general dependence on the ſtate of the ſtomach, in particular, are not more evident, in my opinion, than the relation ſubſiſting between the *senſorium commune* and the internal ſenſes, and the dependence of the latter on the impreſſions made on the nervous ſenſorium, or common receptacle.

The immediate influence of the mind on the nervous ſyſtem, and the agency of the latter in the operations of the mind upon the ſyſtem, render it probable that they are concerned in the formation of that ſympathetic orgaſm which I ſuggeſt as the prediſpoſition to mental diſorder.

The tranſlation of diſeaſes from the blood-veſſels to the brain and nervous ſyſtem, and the transformation of febrile action to mania, alſo evince that phyſical relationſhip ſo frequently mentioned by phyſiologiſts, as connecting mind and body, and ſubjecting each to a participation in the morbid affections of the other. This intimate connection, however, does not invalidate the doctrine of ſeparate orders of idiopathic diſeaſe. Fanaticiſm appears to be as much a primary mental diſorder, as febrile action is a vaſcular diſeaſe.

In three caſes of fever, which occurred laſt autumn, the transformation to mania was complete, and perfectly conſonant with Dr. Ruſh's theory of the maniacal ſtate of fever. In the firſt caſe a remittent form of fever became an intermittent hyſteria. After a ſlight nervous agitation the patient would become frantic, alternately laughing and crying for ſeveral hours. The paroxyſms reſembled intermittent fever in the regularity of recurrence and the term of duration; and were, accordingly, removed in a few days, by the uſe of asafœtida and Peruvian bark in the intermiſſions.

A ſecond caſe of remittent fever terminated on the fifth day, in a moderate ſweat; and the ſixth day I pronounced my patient free from diſeaſe, and recommended a convaleſcent regimen. On the ſeventh day I was called to him again, and, to my great ſurpriſe, found him maniacal, with a feeble and frequent pulſe, and cool ſkin. Suſpecting the change to be a tranſlation of morbid action from the vaſcular ſyſtem to the brain, brain, I endeavoured to relieve the latter by the application of cold water to the head for thirty-six hours, without any effect but an occasional shuddering sensation. Afterwards I attempted a revulsion of excitement by the application of blisters to the scalp, hands and feet, with no better success; and, finally, to excite counter action in the stomach by stimulating draughts. After remaining *in statu quo* for several days, my patient was put under the care of one of Mesmers' disciples, who professed wonders, and promised no less; and with earnest wishes for his better success, and strong apprehensions of disappointment to the relatives, I submitted to the transfer; and the result confirmed my expectations.

A third case, to which I was called in the last stage of the disease, was similar in progress and termination to the preceding.

It is also worthy of remark, that the diseases of last autumn affected the brain and nervous system in an astonishing manner. The most trifling casualties sometimes produced delirium; and insanity and suicide were, throughout the country, frequent beyond example. I have sometimes queried, whether there might not have been an epidemic constitution of atmosphere, resembling the pestilential constitution of the air described by Sydenham, and believed, by many, to have existed in our country for some years past. The *maladie Anglaise* is imputed to the influence of a Novemher atmosphere.

In consequence of Dr. Miller's reasoning on the morbid functions of the stomach, I refer these facts and observations to him; with the simple query, whether the fanaticism of the S— family be accountable for on the sympathetic connection existing between the stomach and nervous system? or, whether it be not exclusively a mental affection?

